# The Domestic Staff—VII

**Published:** 1908-10-17

**Authors:** 


### Nursing Administration.

### THE DOMESTIC STAFF—VII.

### HOW TO REDUCE EXPENDITURE.

One after another the strongholds of prejudice in institution management have given away before the light of modern skill in adapting means to the required end, but domestic service still in many quarters remains in its old rut of unintelligent tradition. It can scarcely be doubted by any who have studied the variations in the cost of service which have been brought under notice in a previous article—and these variations are small compared to those which appear in "Burdett's Annual"—that these variations are not absolutely inevitable. Certainly the administration varies greatly between one hospital and another, but the general routine is much the same in one as in another. If some general hospitals can cut down domestic expenses to the level of from £5 to £8 per bed, while some with medical schools spend no more than £10 or £11, is it not remarkable that other establishments spend from £15 to £20 per bed on the same item? It may be taken for granted that any attempt to reckon the amount which ought to be expended on service, taken *en gros*, must be futile. The question can only profitably be considered in detail in relation to each department of the work, and in all its bearings. The three principal factors in expenditure are numbers, wages and board. Of these items, the question of numbers is, as we have endeavoured to show, the most important. To work the establishment efficiently with the fewest possible hands, one competent person must be placed in charge of each separate section of the domestic service, each of these head workers being subject to the control of the matron or her housekeeper, and having subordinate juniors under her in process of training; and the same principle must be followed out with the male workers. By these means an exact equilibrium can be maintained. There is no overlapping, no interference of one with another; and a direct sense of responsibility rests consciously on all in charge.

It may be surmised that the item of board counts for a good part of the variation observable in the "cost of service." When the board of the domestic staff is not separated from that of the nurses, or even from that of the patients, the estimates made of it by guess-work are often curiously wide of the mark. It would, of course, be quite possible to waste a great deal of money over the board of domestics, but it is doubtful if this often happens. In all probability the weekly average of 6s. a head for females and 7s. a head for males is seldom exceeded, although in some establishments a much lower average is attained.

The question of wages is a very complex one, and cannot profitably be entered into, owing to the different rates paid in different localities and the variety of posts which have to be filled in institutions. It is not good economy to pare down the wages to such an extent that efficiency is discouraged, for it is perfectly certain that inferior rates of pay can only attract inferior workers. Occasionally it may, and does to our knowledge, happen that the hospital offers so strong an attraction to its permanent staff of domestics, that some will remain on in its service at a lower wage than could be obtained elsewhere, rather than leave congenial work or a loved mistress. But neither the exceptional worker nor the exceptional mistress can be counted on in this connection.

In general it will be found that male service is expensive and unsatisfactory as compared with female service. The men who can be obtained for indoor service in institutions are not readily teachable, and their notions of cleanliness are apt to be precarious. They are usually men who have been trained for quite other kinds of work, and it is difficult to get them into line. It must be readily acknowledged, however, that the right kind of factotum in a hospital is an invaluable person. In hospitals with a medical school, undertaking a great variety of work, a large staff of male workers seems to be indispensable. These will include hall porter, out-patients' porters, dispensary porter, theatre porter, engineer (with one or more assistants), stokers, electrician, storekeeper, mortuary attendant, house carpenter, painter or handyman, window cleaners, lift boy, and such other boys or helpers as may be needed to assist the porters, etc., in each department. When the requirements are of a nature to make the service a career for such assistants as enter young, the hospital is in a much better position as regards the male staff, since an opportunity is afforded for training them up from the beginning. From the point of view of wages, male service is indisputably more costly than female. The porter, stoker, and other non-resident men employed will get from 23s. to 30s. a week with or without uniform, or from £60 to £80 a year. Reckoning the value of the board received by the female staff at £15 a year, it will be seen that few of them receive more than £30 a year, and none as much as £40. Very few even of the nursing staff receive the equivalent of £80 a year. Hence, in aiming at economy of service, the most important point of all is to keep down the number of men employed about the establishment. The employment of a large number of men is often a survival of old times before the birth of the training school, and before the discovery of the fact that for the performance of domestic duties, well-trained women are immeasurably more satisfactory than men. The bare enumeration of the various people required to keep a big establishment going is sufficient to show why in large institutions, where work is done at high pressure and every department is filled and emptied by different sets of people several times each day, the cost of service is high. It is by the volume of work accomplished, rather than by the size and number of the wards, that such establishments are compelled to measure their requirements.

